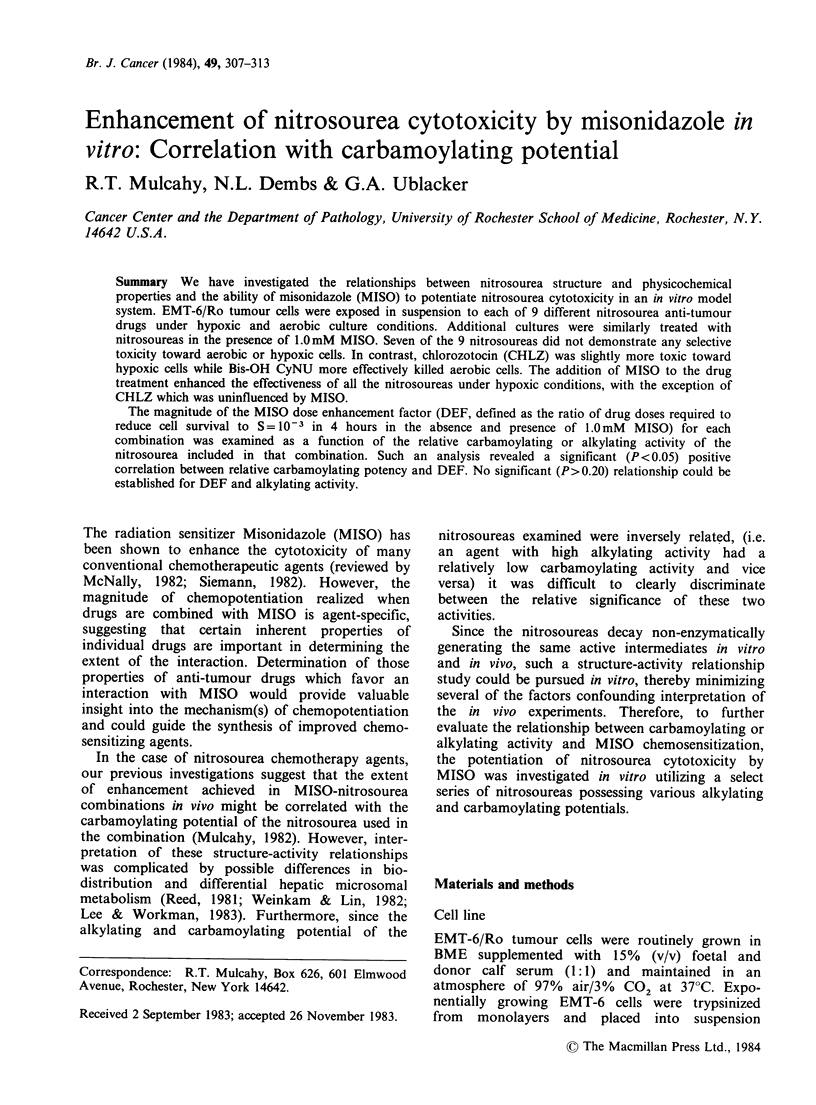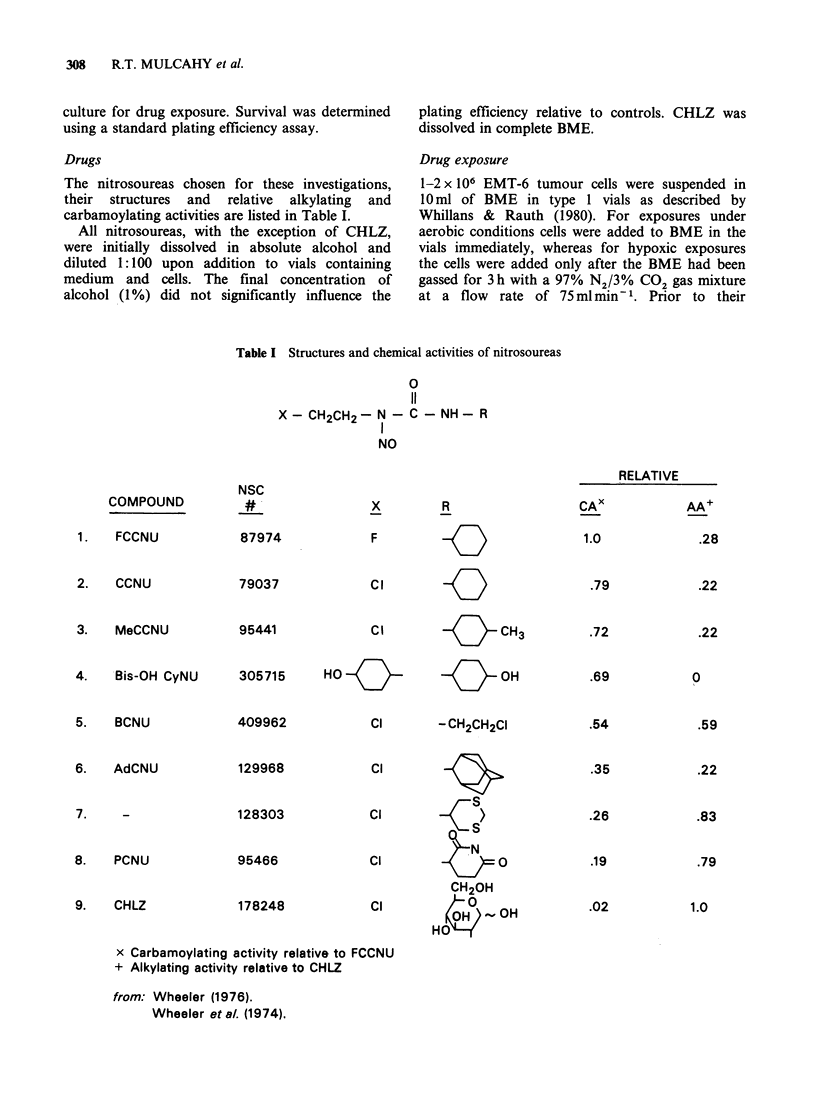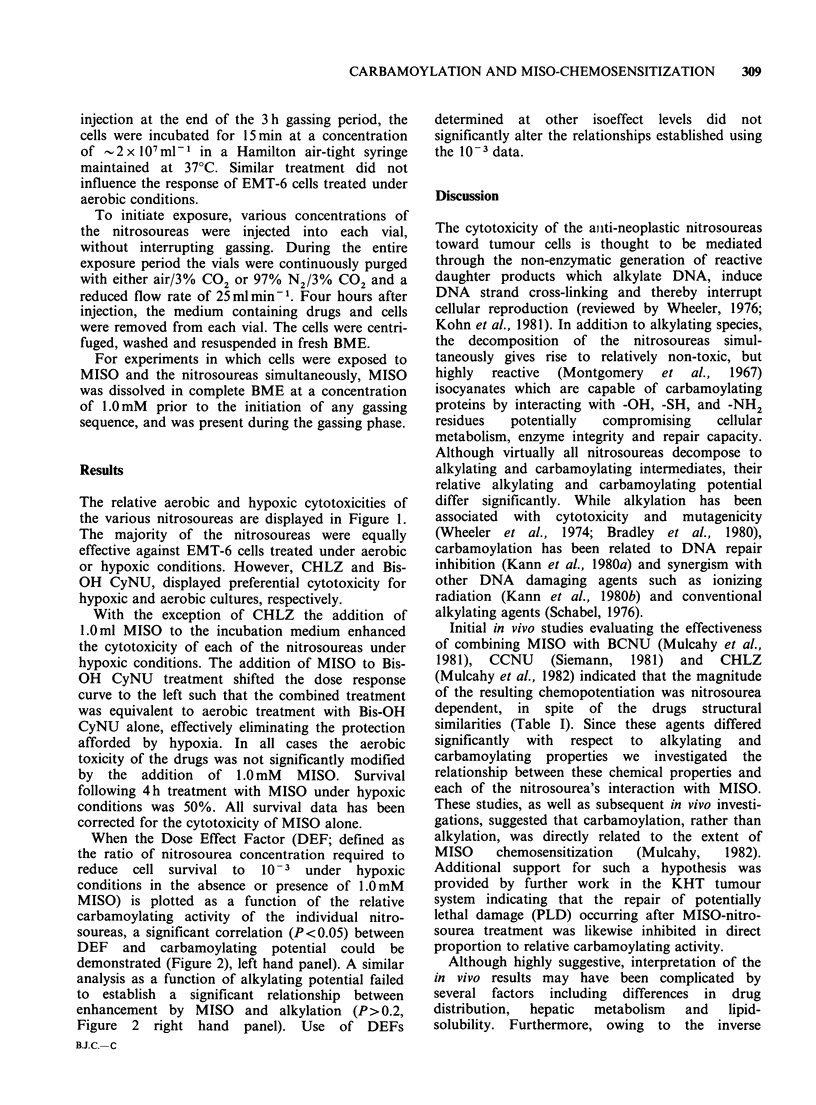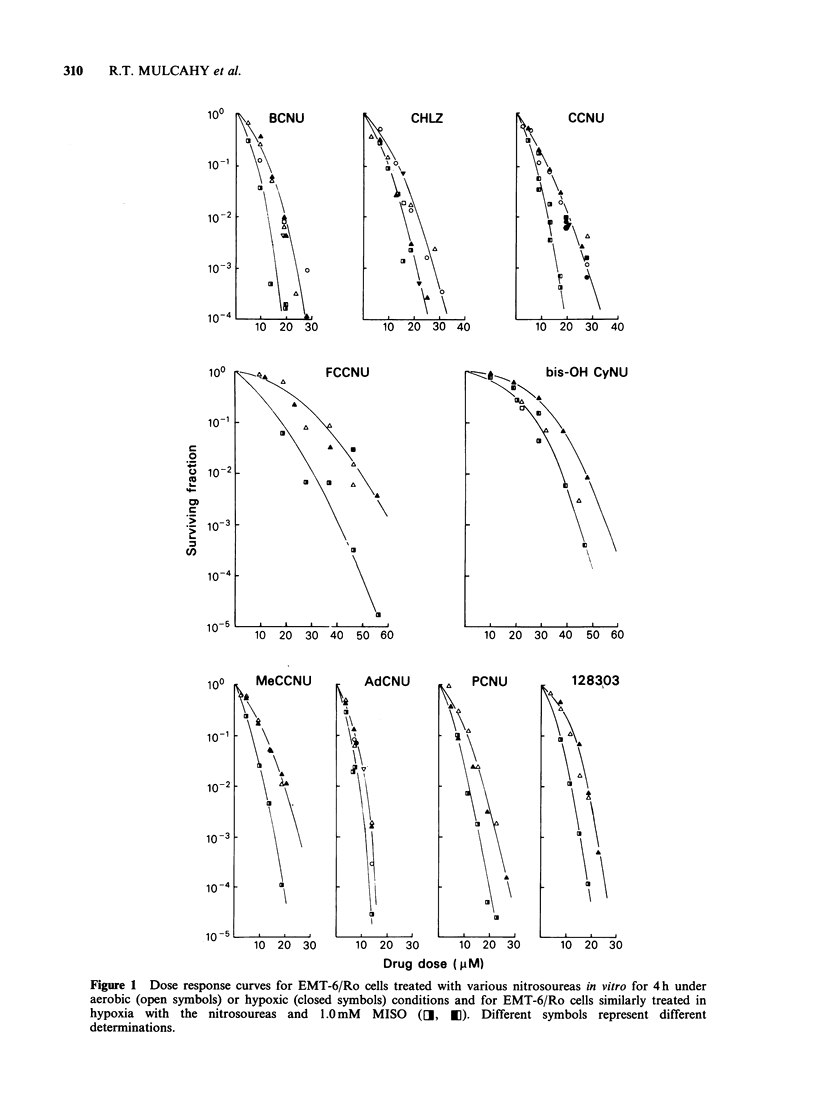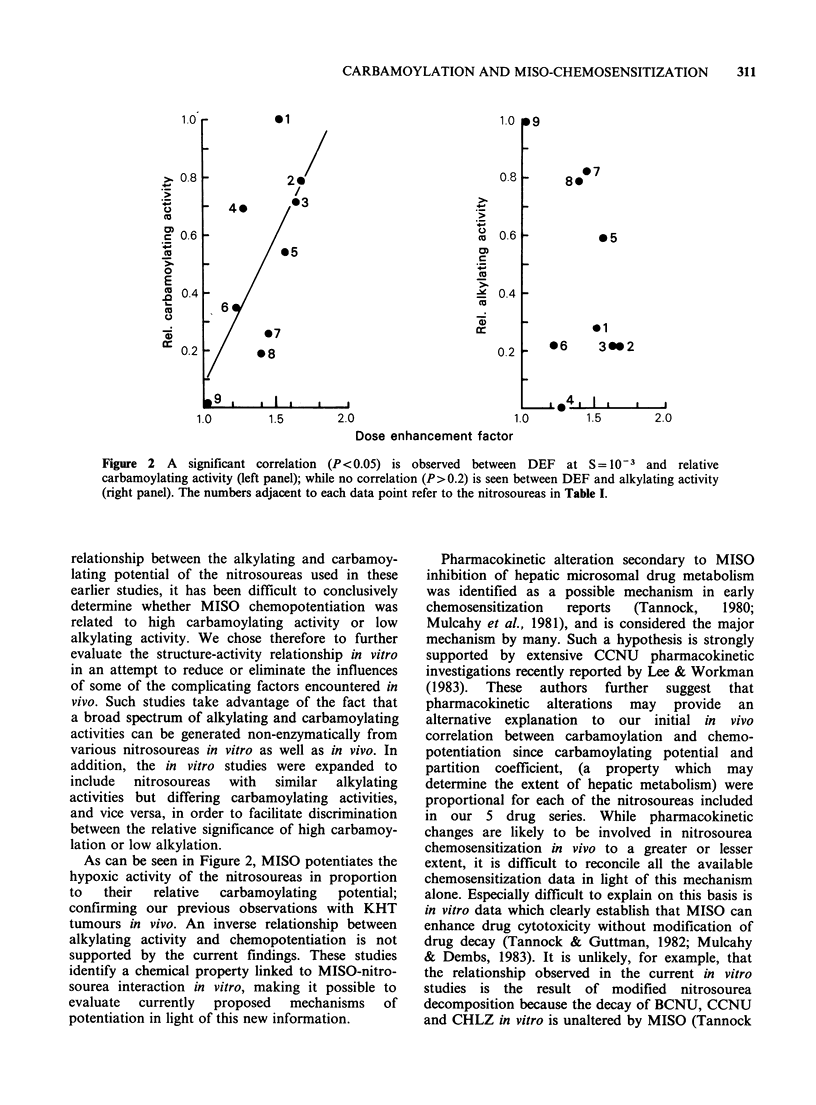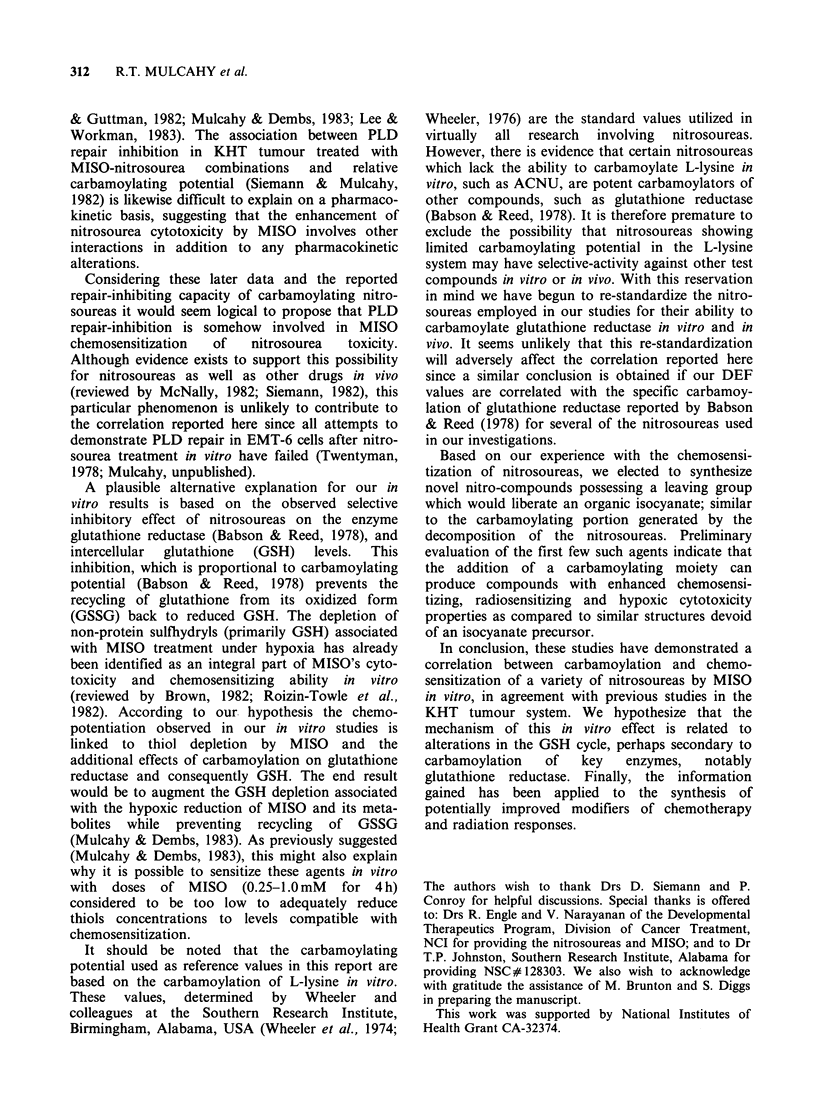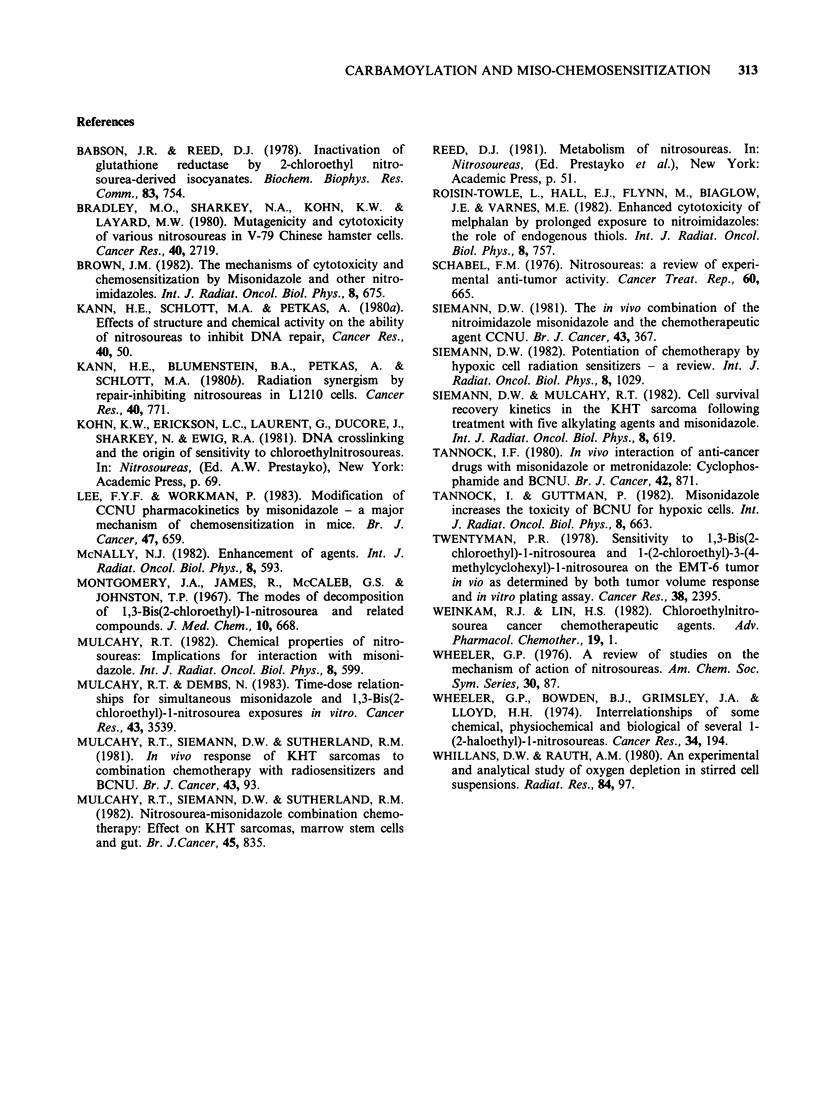# Enhancement of nitrosourea cytotoxicity by misonidazole in vitro: correlation with carbamoylating potential.

**DOI:** 10.1038/bjc.1984.48

**Published:** 1984-03

**Authors:** R. T. Mulcahy, N. L. Dembs, G. A. Ublacker

## Abstract

We have investigated the relationships between nitrosourea structure and physicochemical properties and the ability of misonidazole (MISO) to potentiate nitrosourea cytotoxicity in an in vitro model system. EMT-6/Ro tumour cells were exposed in suspension to each of 9 different nitrosourea anti-tumour drugs under hypoxic and aerobic culture conditions. Additional cultures were similarly treated with nitrosoureas in the presence of 1.0 mM MISO. Seven of the 9 nitrosoureas did not demonstrate any selective toxicity toward aerobic or hypoxic cells. In contrast, chlorozotocin (CHLZ) was slightly more toxic toward hypoxic cells while Bis-OH CyNU more effectively killed aerobic cells. The addition of MISO to the drug treatment enhanced the effectiveness of all the nitrosoureas under hypoxic conditions, with the exception of CHLZ which was uninfluenced by MISO. The magnitude of the MISO dose enhancement factor (DEF, defined as the ratio of drug doses required to reduce cell survival to S = 10(-3) in 4 hours in the absence and presence of 1.0 mM MISO) for each combination was examined as a function of the relative carbamoylating or alkylating activity of the nitrosourea included in that combination. Such an analysis revealed a significant (P less than 0.05) positive correlation between relative carbamoylating potency and DEF. No significant (P greater than 0.20) relationship could be established for DEF and alkylating activity.


*Br. J. Cancer* (1984), **49**, 307–313

### Enhancement of nitrosourea cytotoxicity by misonidazole *in vitro*: Correlation with carbamoylating potential

R.T. Mulcahy, N.L. Dembs & G.A. Ublacker

*Cancer Center and the Department of Pathology, University of Rochester School of Medicine, Rochester, N.Y. 14642 U.S.A.*

**Summary** We have investigated the relationships between nitrosourea structure and physicochemical properties and the ability of misonidazole (MISO) to potentiate nitrosourea cytotoxicity in an *in vitro* model system. EMT-6/Ro tumour cells were exposed in suspension to each of 9 different nitrosourea anti-tumour drugs under hypoxic and aerobic culture conditions. Additional cultures were similarly treated with nitrosoureas in the presence of 1.0 mM MISO. Seven of the 9 nitrosoureas did not demonstrate any selective toxicity toward aerobic or hypoxic cells. In contrast, chlorozotocin (CHLZ) was slightly more toxic toward hypoxic cells while Bis-OH CyNU more effectively killed aerobic cells. The addition of MISO to the drug treatment enhanced the effectiveness of all the nitrosoureas under hypoxic conditions, with the exception of CHLZ which was uninfluenced by MISO.

The magnitude of the MISO dose enhancement factor (DEF, defined as the ratio of drug doses required to reduce cell survival to $S=10^{-3}$ in 4 hours in the absence and presence of 1.0 mM MISO) for each combination was examined as a function of the relative carbamoylating or alkylating activity of the nitrosourea included in that combination. Such an analysis revealed a significant ($P<0.05$) positive correlation between relative carbamoylating potency and DEF. No significant ($P>0.20$) relationship could be established for DEF and alkylating activity.

The radiation sensitizer Misonidazole (MISO) has been shown to enhance the cytotoxicity of many conventional chemotherapeutic agents (reviewed by McNally, 1982; Siemann, 1982). However, the magnitude of chemopotentiation realized when drugs are combined with MISO is agent-specific, suggesting that certain inherent properties of individual drugs are important in determining the extent of the interaction. Determination of those properties of anti-tumour drugs which favor an interaction with MISO would provide valuable insight into the mechanism(s) of chemopotentiation and could guide the synthesis of improved chemosensitizing agents.

In the case of nitrosourea chemotherapy agents, our previous investigations suggest that the extent of enhancement achieved in MISO-nitrosourea combinations *in vivo* might be correlated with the carbamoylating potential of the nitrosourea used in the combination (Mulcahy, 1982). However, interpretation of these structure-activity relationships was complicated by possible differences in biodistribution and differential hepatic microsomal metabolism (Reed, 1981; Weinkam & Lin, 1982; Lee & Workman, 1983). Furthermore, since the alkylating and carbamoylating potential of the nitrosoureas examined were inversely related, (i.e. an agent with high alkylating activity had a relatively low carbamoylating activity and vice versa) it was difficult to clearly discriminate between the relative significance of these two activities.

Correspondence: R.T. Mulcahy, Box 626, 601 Elmwood Avenue, Rochester, New York 14642.

Received 2 September 1983; accepted 26 November 1983.

Since the nitrosoureas decay non-enzymatically generating the same active intermediates *in vitro* and *in vivo*, such a structure-activity relationship study could be pursued *in vitro*, thereby minimizing several of the factors confounding interpretation of the *in vivo* experiments. Therefore, to further evaluate the relationship between carbamoylating or alkylating activity and MISO chemosensitization, the potentiation of nitrosourea cytotoxicity by MISO was investigated *in vitro* utilizing a select series of nitrosoureas possessing various alkylating and carbamoylating potentials.

### Materials and methods

### Cell line

EMT-6/Ro tumour cells were routinely grown in BME supplemented with 15% (v/v) foetal and donor calf serum (1:1) and maintained in an atmosphere of 97% air/3% $CO_2$ at 37°C. Exponentially growing EMT-6 cells were trypsinized from monolayers and placed into suspension

© The Macmillan Press Ltd., 1984

culture for drug exposure. Survival was determined using a standard plating efficiency assay.

*Drugs*

The nitrosoureas chosen for these investigations, their structures and relative alkylating and carbamoylating activities are listed in Table I.

All nitrosoureas, with the exception of CHLZ, were initially dissolved in absolute alcohol and diluted 1:100 upon addition to vials containing medium and cells. The final concentration of alcohol (1%) did not significantly influence the plating efficiency relative to controls. CHLZ was dissolved in complete BME.

*Drug exposure*

$1\text{–}2 \times 10^6$ EMT-6 tumour cells were suspended in 10 ml of BME in type 1 vials as described by Whillans & Rauth (1980). For exposures under aerobic conditions cells were added to BME in the vials immediately, whereas for hypoxic exposures the cells were added only after the BME had been gassed for 3 h with a 97% $N_2$/3% $CO_2$ gas mixture at a flow rate of 75 ml min$^{-1}$. Prior to their

**Table I** Structures and chemical activities of nitrosoureas

$$X - CH_2CH_2 - \underset{\displaystyle NO}{\underset{|}{N}} - \overset{\displaystyle O}{\overset{\|}{C}} - NH - R$$

| | COMPOUND | NSC # | X | R | RELATIVE CA$^{\times}$ | RELATIVE AA$^{+}$ |
|---|---|---|---|---|---|---|
| 1. | FCCNU | 87974 | F |  | 1.0 | .28 |
| 2. | CCNU | 79037 | Cl |  | .79 | .22 |
| 3. | MeCCNU | 95441 | Cl |  | .72 | .22 |
| 4. | Bis-OH CyNU | 305715 |  |  | .69 | 0 |
| 5. | BCNU | 409962 | Cl | $-CH_2CH_2Cl$ | .54 | .59 |
| 6. | AdCNU | 129968 | Cl |  | .35 | .22 |
| 7. | - | 128303 | Cl |  | .26 | .83 |
| 8. | PCNU | 95466 | Cl |  | .19 | .79 |
| 9. | CHLZ | 178248 | Cl |  | .02 | 1.0 |

× Carbamoylating activity relative to FCCNU
+ Alkylating activity relative to CHLZ

*from:* Wheeler (1976).
Wheeler *et al.* (1974).

injection at the end of the 3 h gassing period, the cells were incubated for 15 min at a concentration of $\sim 2 \times 10^7 ml^{-1}$ in a Hamilton air-tight syringe maintained at 37°C. Similar treatment did not influence the response of EMT-6 cells treated under aerobic conditions.

To initiate exposure, various concentrations of the nitrosoureas were injected into each vial, without interrupting gassing. During the entire exposure period the vials were continuously purged with either air/3% $CO_2$ or 97% $N_2$/3% $CO_2$ and a reduced flow rate of $25 ml min^{-1}$. Four hours after injection, the medium containing drugs and cells were removed from each vial. The cells were centrifuged, washed and resuspended in fresh BME.

For experiments in which cells were exposed to MISO and the nitrosoureas simultaneously, MISO was dissolved in complete BME at a concentration of 1.0 mM prior to the initiation of any gassing sequence, and was present during the gassing phase.

### Results

The relative aerobic and hypoxic cytotoxicities of the various nitrosoureas are displayed in Figure 1. The majority of the nitrosoureas were equally effective against EMT-6 cells treated under aerobic or hypoxic conditions. However, CHLZ and Bis-OH CyNU, displayed preferential cytotoxicity for hypoxic and aerobic cultures, respectively.

With the exception of CHLZ the addition of 1.0 ml MISO to the incubation medium enhanced the cytotoxicity of each of the nitrosoureas under hypoxic conditions. The addition of MISO to Bis-OH CyNU treatment shifted the dose response curve to the left such that the combined treatment was equivalent to aerobic treatment with Bis-OH CyNU alone, effectively eliminating the protection afforded by hypoxia. In all cases the aerobic toxicity of the drugs was not significantly modified by the addition of 1.0 mM MISO. Survival following 4 h treatment with MISO under hypoxic conditions was 50%. All survival data has been corrected for the cytotoxicity of MISO alone.

When the Dose Effect Factor (DEF; defined as the ratio of nitrosourea concentration required to reduce cell survival to $10^{-3}$ under hypoxic conditions in the absence or presence of 1.0 mM MISO) is plotted as a function of the relative carbamoylating activity of the individual nitrosoureas, a significant correlation ($P < 0.05$) between DEF and carbamoylating potential could be demonstrated (Figure 2), left hand panel). A similar analysis as a function of alkylating potential failed to establish a significant relationship between enhancement by MISO and alkylation ($P > 0.2$, Figure 2 right hand panel). Use of DEFs determined at other isoeffect levels did not significantly alter the relationships established using the $10^{-3}$ data.

### Discussion

The cytotoxicity of the anti-neoplastic nitrosoureas toward tumour cells is thought to be mediated through the non-enzymatic generation of reactive daughter products which alkylate DNA, induce DNA strand cross-linking and thereby interrupt cellular reproduction (reviewed by Wheeler, 1976; Kohn *et al.*, 1981). In addition to alkylating species, the decomposition of the nitrosoureas simultaneously gives rise to relatively non-toxic, but highly reactive (Montgomery *et al.*, 1967) isocyanates which are capable of carbamoylating proteins by interacting with -OH, -SH, and $-NH_2$ residues potentially compromising cellular metabolism, enzyme integrity and repair capacity. Although virtually all nitrosoureas decompose to alkylating and carbamoylating intermediates, their relative alkylating and carbamoylating potential differ significantly. While alkylation has been associated with cytotoxicity and mutagenicity (Wheeler *et al.*, 1974; Bradley *et al.*, 1980), carbamoylation has been related to DNA repair inhibition (Kann *et al.*, 1980*a*) and synergism with other DNA damaging agents such as ionizing radiation (Kann *et al.*, 1980*b*) and conventional alkylating agents (Schabel, 1976).

Initial *in vivo* studies evaluating the effectiveness of combining MISO with BCNU (Mulcahy *et al.*, 1981), CCNU (Siemann, 1981) and CHLZ (Mulcahy *et al.*, 1982) indicated that the magnitude of the resulting chemopotentiation was nitrosourea dependent, in spite of the drugs structural similarities (Table I). Since these agents differed significantly with respect to alkylating and carbamoylating properties we investigated the relationship between these chemical properties and each of the nitrosourea's interaction with MISO. These studies, as well as subsequent *in vivo* investigations, suggested that carbamoylation, rather than alkylation, was directly related to the extent of MISO chemosensitization (Mulcahy, 1982). Additional support for such a hypothesis was provided by further work in the KHT tumour system indicating that the repair of potentially lethal damage (PLD) occurring after MISO-nitrosourea treatment was likewise inhibited in direct proportion to relative carbamoylating activity.

Although highly suggestive, interpretation of the *in vivo* results may have been complicated by several factors including differences in drug distribution, hepatic metabolism and lipid-solubility. Furthermore, owing to the inverse

**Figure 1** Dose response curves for EMT-6/Ro cells treated with various nitrosoureas *in vitro* for 4h under aerobic (open symbols) or hypoxic (closed symbols) conditions and for EMT-6/Ro cells similarly treated in hypoxia with the nitrosoureas and 1.0mM MISO (◨, ◧). Different symbols represent different determinations.

**Figure 2** A significant correlation ($P<0.05$) is observed between DEF at $S=10^{-3}$ and relative carbamoylating activity (left panel); while no correlation ($P>0.2$) is seen between DEF and alkylating activity (right panel). The numbers adjacent to each data point refer to the nitrosoureas in **Table I**.

relationship between the alkylating and carbamoylating potential of the nitrosoureas used in these earlier studies, it has been difficult to conclusively determine whether MISO chemopotentiation was related to high carbamoylating activity or low alkylating activity. We chose therefore to further evaluate the structure-activity relationship *in vitro* in an attempt to reduce or eliminate the influences of some of the complicating factors encountered *in vivo*. Such studies take advantage of the fact that a broad spectrum of alkylating and carbamoylating activities can be generated non-enzymatically from various nitrosoureas *in vitro* as well as *in vivo*. In addition, the *in vitro* studies were expanded to include nitrosoureas with similar alkylating activities but differing carbamoylating activities, and vice versa, in order to facilitate discrimination between the relative significance of high carbamoylation or low alkylation.

As can be seen in Figure 2, MISO potentiates the hypoxic activity of the nitrosoureas in proportion to their relative carbamoylating potential; confirming our previous observations with KHT tumours *in vivo*. An inverse relationship between alkylating activity and chemopotentiation is not supported by the current findings. These studies identify a chemical property linked to MISO-nitrosourea interaction *in vitro*, making it possible to evaluate currently proposed mechanisms of potentiation in light of this new information.

Pharmacokinetic alteration secondary to MISO inhibition of hepatic microsomal drug metabolism was identified as a possible mechanism in early chemosensitization reports (Tannock, 1980; Mulcahy *et al.*, 1981), and is considered the major mechanism by many. Such a hypothesis is strongly supported by extensive CCNU pharmacokinetic investigations recently reported by Lee & Workman (1983). These authors further suggest that pharmacokinetic alterations may provide an alternative explanation to our initial *in vivo* correlation between carbamoylation and chemopotentiation since carbamoylating potential and partition coefficient, (a property which may determine the extent of hepatic metabolism) were proportional for each of the nitrosoureas included in our 5 drug series. While pharmacokinetic changes are likely to be involved in nitrosourea chemosensitization *in vivo* to a greater or lesser extent, it is difficult to reconcile all the available chemosensitization data in light of this mechanism alone. Especially difficult to explain on this basis is *in vitro* data which clearly establish that MISO can enhance drug cytotoxicity without modification of drug decay (Tannock & Guttman, 1982; Mulcahy & Dembs, 1983). It is unlikely, for example, that the relationship observed in the current *in vitro* studies is the result of modified nitrosourea decomposition because the decay of BCNU, CCNU and CHLZ *in vitro* is unaltered by MISO (Tannock

& Guttman, 1982; Mulcahy & Dembs, 1983; Lee & Workman, 1983). The association between PLD repair inhibition in KHT tumour treated with MISO-nitrosourea combinations and relative carbamoylating potential (Siemann & Mulcahy, 1982) is likewise difficult to explain on a pharmacokinetic basis, suggesting that the enhancement of nitrosourea cytotoxicity by MISO involves other interactions in addition to any pharmacokinetic alterations.

Considering these later data and the reported repair-inhibiting capacity of carbamoylating nitrosoureas it would seem logical to propose that PLD repair-inhibition is somehow involved in MISO chemosensitization of nitrosourea toxicity. Although evidence exists to support this possibility for nitrosoureas as well as other drugs *in vivo* (reviewed by McNally, 1982; Siemann, 1982), this particular phenomenon is unlikely to contribute to the correlation reported here since all attempts to demonstrate PLD repair in EMT-6 cells after nitrosourea treatment *in vitro* have failed (Twentyman, 1978; Mulcahy, unpublished).

A plausible alternative explanation for our *in vitro* results is based on the observed selective inhibitory effect of nitrosoureas on the enzyme glutathione reductase (Babson & Reed, 1978), and intercellular glutathione (GSH) levels. This inhibition, which is proportional to carbamoylating potential (Babson & Reed, 1978) prevents the recycling of glutathione from its oxidized form (GSSG) back to reduced GSH. The depletion of non-protein sulfhydryls (primarily GSH) associated with MISO treatment under hypoxia has already been identified as an integral part of MISO's cytotoxicity and chemosensitizing ability *in vitro* (reviewed by Brown, 1982; Roizin-Towle *et al.*, 1982). According to our hypothesis the chemopotentiation observed in our *in vitro* studies is linked to thiol depletion by MISO and the additional effects of carbamoylation on glutathione reductase and consequently GSH. The end result would be to augment the GSH depletion associated with the hypoxic reduction of MISO and its metabolites while preventing recycling of GSSG (Mulcahy & Dembs, 1983). As previously suggested (Mulcahy & Dembs, 1983), this might also explain why it is possible to sensitize these agents *in vitro* with doses of MISO (0.25–1.0 mM for 4 h) considered to be too low to adequately reduce thiols concentrations to levels compatible with chemosensitization.

It should be noted that the carbamoylating potential used as reference values in this report are based on the carbamoylation of L-lysine *in vitro*. These values, determined by Wheeler and colleagues at the Southern Research Institute, Birmingham, Alabama, USA (Wheeler *et al.*, 1974; Wheeler, 1976) are the standard values utilized in virtually all research involving nitrosoureas. However, there is evidence that certain nitrosoureas which lack the ability to carbamoylate L-lysine *in vitro*, such as ACNU, are potent carbamoylators of other compounds, such as glutathione reductase (Babson & Reed, 1978). It is therefore premature to exclude the possibility that nitrosoureas showing limited carbamoylating potential in the L-lysine system may have selective-activity against other test compounds *in vitro* or *in vivo*. With this reservation in mind we have begun to re-standardize the nitrosoureas employed in our studies for their ability to carbamoylate glutathione reductase *in vitro* and *in vivo*. It seems unlikely that this re-standardization will adversely affect the correlation reported here since a similar conclusion is obtained if our DEF values are correlated with the specific carbamoylation of glutathione reductase reported by Babson & Reed (1978) for several of the nitrosoureas used in our investigations.

Based on our experience with the chemosensitization of nitrosoureas, we elected to synthesize novel nitro-compounds possessing a leaving group which would liberate an organic isocyanate; similar to the carbamoylating portion generated by the decomposition of the nitrosoureas. Preliminary evaluation of the first few such agents indicate that the addition of a carbamoylating moiety can produce compounds with enhanced chemosensitizing, radiosensitizing and hypoxic cytotoxicity properties as compared to similar structures devoid of an isocyanate precursor.

In conclusion, these studies have demonstrated a correlation between carbamoylation and chemosensitization of a variety of nitrosoureas by MISO *in vitro*, in agreement with previous studies in the KHT tumour system. We hypothesize that the mechanism of this *in vitro* effect is related to alterations in the GSH cycle, perhaps secondary to carbamoylation of key enzymes, notably glutathione reductase. Finally, the information gained has been applied to the synthesis of potentially improved modifiers of chemotherapy and radiation responses.

The authors wish to thank Drs D. Siemann and P. Conroy for helpful discussions. Special thanks is offered to: Drs R. Engle and V. Narayanan of the Developmental Therapeutics Program, Division of Cancer Treatment, NCI for providing the nitrosoureas and MISO; and to Dr T.P. Johnston, Southern Research Institute, Alabama for providing NSC#128303. We also wish to acknowledge with gratitude the assistance of M. Brunton and S. Diggs in preparing the manuscript.

This work was supported by National Institutes of Health Grant CA-32374.

### References

BABSON, J.R. & REED, D.J. (1978). Inactivation of glutathione reductase by 2-chloroethyl nitrosourea-derived isocyanates. *Biochem. Biophys. Res. Comm.*, **83**, 754.

BRADLEY, M.O., SHARKEY, N.A., KOHN, K.W. & LAYARD, M.W. (1980). Mutagenicity and cytotoxicity of various nitrosoureas in V-79 Chinese hamster cells. *Cancer Res.*, **40**, 2719.

BROWN, J.M. (1982). The mechanisms of cytotoxicity and chemosensitization by Misonidazole and other nitroimidazoles. *Int. J. Radiat. Oncol. Biol. Phys.*, **8**, 675.

KANN, H.E., SCHLOTT, M.A. & PETKAS, A. (1980*a*). Effects of structure and chemical activity on the ability of nitrosoureas to inhibit DNA repair, *Cancer Res.*, **40**, 50.

KANN, H.E., BLUMENSTEIN, B.A., PETKAS, A. & SCHLOTT, M.A. (1980*b*). Radiation synergism by repair-inhibiting nitrosoureas in L1210 cells. *Cancer Res.*, **40**, 771.

KOHN, K.W., ERICKSON, L.C., LAURENT, G., DUCORE, J., SHARKEY, N. & EWIG, R.A. (1981). DNA crosslinking and the origin of sensitivity to chloroethylnitrosoureas. In: *Nitrosoureas,* (Ed. A.W. Prestayko), New York: Academic Press, p. 69.

LEE, F.Y.F. & WORKMAN, P. (1983). Modification of CCNU pharmacokinetics by misonidazole – a major mechanism of chemosensitization in mice. *Br. J. Cancer*, **47**, 659.

McNALLY, N.J. (1982). Enhancement of agents. *Int. J. Radiat. Oncol. Biol. Phys.*, **8**, 593.

MONTGOMERY, J.A., JAMES, R., McCALEB, G.S. & JOHNSTON, T.P. (1967). The modes of decomposition of 1,3-Bis(2-chloroethyl)-1-nitrosourea and related compounds. *J. Med. Chem.*, **10**, 668.

MULCAHY, R.T. (1982). Chemical properties of nitrosoureas: Implications for interaction with misonidazole. *Int. J. Radiat. Oncol. Biol. Phys.*, **8**, 599.

MULCAHY, R.T. & DEMBS, N. (1983). Time-dose relationships for simultaneous misonidazole and 1,3-Bis(2-chloroethyl)-1-nitrosourea exposures *in vitro*. *Cancer Res.*, **43**, 3539.

MULCAHY, R.T., SIEMANN, D.W. & SUTHERLAND, R.M. (1981). *In vivo* response of KHT sarcomas to combination chemotherapy with radiosensitizers and BCNU. *Br. J. Cancer*, **43**, 93.

MULCAHY, R.T., SIEMANN, D.W. & SUTHERLAND, R.M. (1982). Nitrosourea-misonidazole combination chemotherapy: Effect on KHT sarcomas, marrow stem cells and gut. *Br. J.Cancer*, **45**, 835.

REED, D.J. (1981). Metabolism of nitrosoureas. In: *Nitrosoureas*, (Ed. Prestayko *et al.*), New York: Academic Press, p. 51.

ROISIN-TOWLE, L., HALL, E.J., FLYNN, M., BIAGLOW, J.E. & VARNES, M.E. (1982). Enhanced cytotoxicity of melphalan by prolonged exposure to nitroimidazoles: the role of endogenous thiols. *Int. J. Radiat. Oncol. Biol. Phys.*, **8**, 757.

SCHABEL, F.M. (1976). Nitrosoureas: a review of experimental anti-tumor activity. *Cancer Treat. Rep.*, **60**, 665.

SIEMANN, D.W. (1981). The *in vivo* combination of the nitroimidazole misonidazole and the chemotherapeutic agent CCNU. *Br. J. Cancer*, **43**, 367.

SIEMANN, D.W. (1982). Potentiation of chemotherapy by hypoxic cell radiation sensitizers – a review. *Int. J. Radiat. Oncol. Biol. Phys.*, **8**, 1029.

SIEMANN, D.W. & MULCAHY, R.T. (1982). Cell survival recovery kinetics in the KHT sarcoma following treatment with five alkylating agents and misonidazole. *Int. J. Radiat. Oncol. Biol. Phys.*, **8**, 619.

TANNOCK, I.F. (1980). *In vivo* interaction of anti-cancer drugs with misonidazole or metronidazole: Cyclophosphamide and BCNU. *Br. J. Cancer*, **42**, 871.

TANNOCK, I. & GUTTMAN, P. (1982). Misonidazole increases the toxicity of BCNU for hypoxic cells. *Int. J. Radiat. Oncol. Biol. Phys.*, **8**, 663.

TWENTYMAN, P.R. (1978). Sensitivity to 1,3-Bis(2-chloroethyl)-1-nitrosourea and 1-(2-chloroethyl)-3-(4-methylcyclohexyl)-1-nitrosourea on the EMT-6 tumor *in vio* as determined by both tumor volume response and *in vitro* plating assay. *Cancer Res.*, **38**, 2395.

WEINKAM, R.J. & LIN, H.S. (1982). Chloroethylnitrosourea cancer chemotherapeutic agents. *Adv. Pharmacol. Chemother.*, **19**, 1.

WHEELER, G.P. (1976). A review of studies on the mechanism of action of nitrosoureas. *Am. Chem. Soc. Sym. Series*, **30**, 87.

WHEELER, G.P., BOWDEN, B.J., GRIMSLEY, J.A. & LLOYD, H.H. (1974). Interrelationships of some chemical, physiochemical and biological of several 1-(2-haloethyl)-1-nitrosoureas. *Cancer Res.*, **34**, 194.

WHILLANS, D.W. & RAUTH, A.M. (1980). An experimental and analytical study of oxygen depletion in stirred cell suspensions. *Radiat. Res.*, **84**, 97.